# Kratom Abuse Potential 2021: An Updated Eight Factor Analysis

**DOI:** 10.3389/fphar.2021.775073

**Published:** 2022-01-28

**Authors:** Jack E. Henningfield, Daniel W. Wang, Marilyn A. Huestis

**Affiliations:** PinneyAssociates, Inc., Bethesda, MD, United States

**Keywords:** dietary supplement, safety, abuse potential, epidemiology, substance use disorder treatment, opioid pharmacology, Controlled Substances Act

## Abstract

Drugs are regulated in the United States (US) by the Controlled Substances Act (CSA) if assessment of their abuse potential, including public health risks, show such control is warranted. An evaluation via the 8 factors of the CSA provides the comprehensive assessment required for permanent listing of new chemical entities and previously uncontrolled substances. Such an assessment was published for two kratom alkaloids in 2018 that the Food and Drug Administration (FDA) have identified as candidates for CSA listing: mitragynine (MG) and 7-hydroxymitragynine (7-OH-MG) (Henningfield et al., 2018a). That assessment concluded the abuse potential of MG was within the range of many other uncontrolled substances, that there was not evidence of an imminent risk to public health, and that a Schedule I listing (the only option for substances that are not FDA approved for therapeutic use such as kratom) carried public health risks including drug overdoses by people using kratom to abstain from opioids. The purpose of this review is to provide an updated abuse potential assessment reviewing greater than 100 studies published since January 1, 2018. These include studies of abuse potential and physical dependence/withdrawal in animals; *in-vitro* receptor binding; assessments of potential efficacy treating pain and substance use disorders; pharmacokinetic/pharmacodynamic studies with safety-related findings; clinical studies of long-term users with various physiological endpoints; and surveys of patterns and reasons for use and associated effects including dependence and withdrawal. Findings from these studies suggest that public health is better served by assuring continued access to kratom products by consumers and researchers. Currently, Kratom alkaloids and derivatives are in development as safer and/or more effective medicines for treating pain, substances use disorders, and mood disorders. Placing kratom in the CSA via scheduling would criminalize consumers and possession, seriously impede research, and can be predicted to have serious adverse public health consequences, including potentially thousands of drug overdose deaths. Therefore, CSA listing is not recommended. Regulation to minimize risks of contaminated, adulterated, and inappropriately marketed products is recommended.

## 1 Introduction

This is an update to the Henningfield et al. (2018) assessment of the abuse potential of kratom based on the eight factors of the United States Controlled Substances Act (US CSA) (Henningfield et al., 2018a) and summarizes new scientific findings from January 2018 through August 2021. The CSA eight factors evaluate pharmacological actions in the brain or central nervous system (CNS) that may lead to dependence, substance use disorders, or addictions (American Psychiatric Association, 2013; National Institute on Drug Abuse, 2019; World Health Organization, 1994; O’Brien et al., 2011). Abuse potential assessments determine whether substances and medicinal products should be controlled by the CSA (scheduled), and if so the restrictiveness or level of control. Substances are only placed in Schedule I (heroin, LSD, cannabis) when there is no FDA approved therapeutic use and sufficient abuse potential to merit control. Substances with approved therapeutic uses and sufficient abuse potential must be placed in Schedules II–V. By law, an eight-factor analysis (8-FA) provides the primary pharmacological and public health basis for drug scheduling (Food and Drug Administration, 2017a; Belouin and Henningfield, 2018; Johnson et al., 2018). This assessment focuses on kratom and its alkaloids, in particular mitragynine (MG), the primary alkaloid in kratom present in sufficient amounts to account for its effects.

Kratom and its alkaloids are not approved for any therapeutic use by the FDA, are not federally controlled in the US, nor in the International Drug Control Conventions; however some countries do control kratom and/or its two primary alkaloids, MG and 7-OH-MG (Prozialeck et al., 2019; International Narcotics C, 2020a; International Narcotics C, 2020b). Six states in the US (Alabama, Arkansas, Indiana, Tennessee, Vermont and Wisconsin) have banned kratom, while five have passed consumer protection legislation to ensure consumer access to kratom with a framework for regulatory oversight (Arizona, Georgia, Nevada, Oklahoma and Utah). Maryland rejected a proposed ban and passed a minimum age of purchase law (age 21), and at this writing, several states are considering their own kratom consumer protection laws to ensure consumer access but with regulatory oversight. In 2021, Thailand decriminalized kratom farming, possession and sales. In December, 2021, the World Health Organization Expert Committee on Drug Dependence concluded “there is insufficient evidence to recommend a critical review of kratom mitragynine and 7-hydroxymitragynine” [for potential scheduling] but should be kept under surveillance (Commission on Narcotic Drugs, 2021).

In August 2016 the US Drug Enforcement Agency (DEA) proposed scheduling kratom on a temporary “emergency” basis but withdrew the proposal due to thousands of comments from kratom consumers and bipartisan members of Congress, and out of concern that people who were managing their opioid use disorder with the aid of kratom would return to opioid use. The DEA requested that FDA perform a full 8-FA and develop its own independent recommendations related to scheduling (Ingraham, 2016a; Ingraham, 2016b). Subsequently, Dr. Henningfield and his colleagues at PinneyAssociates were commissioned by the American Kratom Association’s legal regulatory counsel to develop an 8-FA (Pinney Associates (2016)) for submission to DEA by December 2, 2016. In November 2017, FDA Commissioner Scott Gottlieb announced that kratom carried “narcotic like” risks of addiction and death but did not make public its October 17th recommendation to DEA to permanently place MG and 7-OH-MG in Schedule I of the CSA (Food and Drug Administration, 2017b; Food and Drug Administration, 2017c).

DEA typically responds to formal 8-FA scheduling requests within 90 days, though there is no legal timeline; however, a formal scheduling rescission order was issued on August 18, 2018 from the Assistant Secretary of Health, US Department of Health and Human Services (DHHS) (Giroir, 2018). The order included conclusions based on a DHHS review consistent with those of the Henningfield et al. (2018) 8-FA (Henningfield et al., 2018a). The DHHS rescission letter stated “mitragynine does not satisfy the first of the three statutory requisites for Schedule I”; “There is still debate among reputable scientists over whether kratom by itself is associated with fatal overdoses”; and “there is a significant risk of immediate adverse public health consequences for potentially millions of users if kratom or its components are included in Schedule I.” The letter also raised concerns about “the stifling effect of classification in Schedule l on critical research needed on the complex and potentially useful chemistry of components of kratom.” This letter was not made public until January 2021.

In 2017, the National Institute on Drug Abuse (NIDA) substantially increased its active research program on kratom’s alkaloids and derivatives as potentially safer and less abusable medicines for pain and addiction and other disorders. The purpose of this review is to provide an update of our 2018 article on the abuse potential of kratom. It includes more than 100 new studies related to kratom abuse potential, safety, patterns of use, and potential therapeutic and public health benefits.

## 2 Methods

The intent was to include all new studies published in English relevant to kratom abuse potential, safety and mechanisms of action published in since January 1, 2018 with some essential earlier studies mentioned and referenced to our 2018 review.^1^ This was by comprehensive online literature searches, and direct requests to leading kratom researchers worldwide. To be concise, factors 4, 5, and 6 are considered a single group of public health related factors.^2^ (Henningfield et al., 2018a; Johnson et al., 2018). Factor 8 is unchanged as neither kratom nor its constituents are scheduled.

## 3 Results

### 3.1 Factor 1: Actual or Relative Potential for Abuse

A summary of the references used, along with main findings and comments from the authors of this review are included in Table 1.

**TABLE 1:** Summary of references.

Factor/Description	Citations	Main findings	Comments
Factor 1: Actual or relative potential for abuse			
Intravenous Self-Administration (IV SA)	(Prozialeck et al., 2019), (Grundmann et al., 2018; Yue et al., 2018; Coe et al., 2019; Hemby et al., 2019; Garcia-Romeu et al., 2020)	No evidence of reward	MG pretreatment reduced morphine self-administration
Intracranial Self-Stimulation (ICSS)	(Negus and Miller, 2014)-(Behnood-Rod et al., 2020)	No evidence of reward for MG or 7-OH-MG	
Drug Discrimination	(Hiranita et al., 2020; Reeve et al., 2020; Obeng et al., 2021)	MG showed partial generalization to multiple drugs, including morphine	Strongest generalization of MG was to unscheduled drugs: phenylephrine and lofexidine
		7-OH-MG showed full generalization to morphine	
Conditioned Place Preference (CPP)	(Yusoff et al., 2018; Vijeepallam et al., 2019; Wilson et al., 2020; Japarin et al., 2021)	Mixed evidence of CPP	
Physical Dependence/Withdrawal	(Harun et al., 2020; Hassan et al., 2020; Johari et al., 2021; Hassan et al., 2021; Hassan et al., 20211778; Harun et al., 2021a)	Mixed evidence of weak withdrawal across studies relative to morphine	MG reduces morphine withdrawal and differs from morphine withdrawal on some measures
Survey Data	(Prozialeck et al., 2019), (Grundmann et al., 2018; Coe et al., 2019; Garcia-Romeu et al., 2020), (Singh et al., 2014; Galbis-Reig, 2016; Swogger and Walsh, 2018; Smith et al., 2019; Harun et al., 2021b)	Majority use is for health benefits, not recreational use or to get high. Use is almost exclusively oral, without the tendency of many recreational substance to smoke, inject, and/or nasally insufflate	Most people reporting “kratom addiction” found it weaker and more tolerable and acceptable than “drug” addiction and were more likely so use it to manage other addictions than to use addictively
Factor 2: Scientific evidence of pharmacological effect			
Potential Therapeutic Effects	(Behnood-Rod et al., 2020; Obeng et al., 2021), (Vicknasingam et al., 2020; Chakraborty et al., 2021a)	Kratom’s antinociceptive effects appear to be mediated at least partly by 7-OH-MG metabolite formation	Animal study findings are consistent for use to manage opioid use disorder and withdrawal, pain and suggest exploration for other disorders
Mechanisms of Action	(Prozialeck et al., 2019), (Behnood-Rod et al., 2020), (Hassan et al., 2019; Hiranita et al., 2019; Kruegel et al., 2019; Gutridge et al., 2020; Todd et al., 2020; Suhaimi et al., 2021)	Kratom alkaloids, including 7-OH-MG may interact with opioid receptors, but do not recruit β-arrestin 2	These are consistent with little or no respiratory depression across a broad range of doses and conditions
Kratom Minor Alkaloids and Metabolites	(Kruegel et al., 2019; Chakraborty et al., 2021a; León et al., 2021; Sharma and McCurdy, 2021), (Newman and Cragg, 2016; Sharma et al., 2019; Domnic et al., 2021a; Domnic et al., 2021b; Chear et al., 2021)	Most minor kratom alkaloids and metabolites are in de minimis levels	Some minor alkaloids might influence kratom’s pharmacological effects and merit evaluation for potential therapeutic uses at much higher doses than provided by kratom
Metabolism and Metabolite Profiling	(Kamble et al., 2019; Kamble et al., 2020a; Kamble et al., 2020b)	7-OH-MG appears to metabolize differently in humans than in other species (e.g., rats, dogs, monkeys)	Animal models for kratom alone may not be fully predictive of human effects
Factor 3: Current state of scientific knowledge			
MG and 7-OH-MG PK/PD	(Hiranita et al., 2020), (Avery et al., 2019; Jagabalan et al., 2019; Maxwell et al., 2020)	Greater exposure observed with natural kratom formulations than with oral MG	
Minor Alkaloids PK/PD	(King et al., 2020; Berthold et al., 2021; Kamble et al., 2021)	Approximately one third of minor alkaloids are characterized	
Clinical Studies	(Singh et al., 2018a; Singh et al., 2018b; Singh et al., 2019a; Singh et al., 2020a; Leong Bin Abdullah et al., 2020; Leong Abdullah et al., 2021)	Long term users of kratom have no significant differences in most physiological measures compared to nonusers	These should not be considered definitive safety data but provide a foundation for further studies
Factors 4, 5, and 6—History and Current Patterns of Abuse; The Scope, Significance and Duration of abuse; What, if any, Risk is there to the Public Health			
U.S. National and Federal Survey Data	(National Institute on Drug Abuse, 2019), (Coe et al., 2019)-(Garcia-Romeu et al., 2020), (U.S. Department of health and Human Services, 2020; Schimmel et al., 2021; Covvey et al., 2020; Grundmann, 2017; Drug Abuse Warning Network, 2020; Drug Enforcement Adm, 2020a; Substance Abuse and Menta, 2020; Drug Enforcement Adm, 2020b; Grundmann et al., 2021; Miech et al., 2021)	NSDUH Lifetime Use: 1.4%; Past Year Use 0.7%. Little evidence of use on other federal surveys either because kratom was not specifically included or did not meet the threshold for reporting	Federal survey data provide no evidence that kratom poses an imminent threat to public health but merits continuing monitoring to better understand trends in use
Kratom Use Prevalence	(U.S. Department of Health and Human Services, 2020; Schimmel et al., 2021; Covvey et al., 2020), (Botanical Education Alliance, 2016)	Estimates range from 1.8 million to over 16 million users in the US	It appears likely that there are at least 10 million kratom users in the US but more definitive studies are needed
Kratom Use Associated Mortality	(National Institute on Drug Abuse, 2019), (Giroir, 2018), (Food and Drug Admini, 2018; Gershman et al., 2019; Henningfield et al., 2019; Olsen et al., 2019)	Risk of kratom associated death appears to be at least a thousand times lower than for morphine-like opioids	Approximately 80% of kratom positive or “involved” deaths also detected other drugs of abuse or the decedent had a history of substance use disorders in one study contribution by other drugs not possible to rule out
Mortality Risks Projected as a Result of Banning Licit Kratom	(Henningfield et al., 2018a), (Ingraham, 2016b), (Giroir, 2018), (Grundmann et al., 2018; Coe et al., 2019; Garcia-Romeu et al., 2020), (Grundmann, 2017), (Henningfield et al., 2018b; Henningfield et al., 2018c; Henningfield et al., 2018d; Prozialeck et al., 2020)	Surveys suggest that it is likely that some kratom users would return to opioid use if kratom use and possession is banned	Fears of relapse to opioid use was a serious concern voiced by thousands of users in surveys and comments to DEA and FDA
Public Health and Individual Benefits of Kratom	(Henningfield et al., 2018a), (Prozialeck et al., 2019), (Coe et al., 2019)-(Garcia-Romeu et al., 2020), (Swogger and Walsh, 2018), (Grundmann, 2017), (Drug Enforcement Adm, 2016), (Raffa, 2014)-(Pain News Network, 2018)	Kratom is used by millions of people in the US to manage substance use disorders, pain, mood disorder, and for energy and improved mental focus and alertness	Reasons for use of kratom rather than FDA approved medications included better efficacy, presumed lower risks and because it is more accessible and tolerable, and/or preferred as a “natural product”. Note: such data should not be used to support therapeutic claims in labeling or marketing
Kratom Use for Managing Opioid Use/Withdrawal and Other Health Reasons	(Coe et al., 2019), (Grundmann, 2017), (Singh et al., 2019b; Singh et al., 2020b; Singh et al., 2020c)	Surveys in US and SEA report kratom is used mostly for its health benefits, including opioid withdrawal	Although management of opioid use and withdrawal is prominent, nonclinical data suggest that use for other substance use disorder management and many other disorders merit further exploration
Comment on Therapeutic Use in Context of FDA Standards	(Katz, 2004; DiMasi et al., 2016; Food and Drug Admini, 2016; Dabrowska and Thaul, 2018; Wouters et al., 2020)	While research has yet to meet FDA’s standard for therapeutic efficacy (NDA), there is substantial evidence of its use and efficacy in treating opioid withdrawal symptoms, and other disorders	
Factor 7—The psychic or physiological dependence liability			
Science Updates	(Hemby et al., 2019), (Coe et al., 2019)-(Garcia-Romeu et al. 2020), (Swogger and Walsh, 2018), (Harun et al., 2021b)-(Vicknasingam et al., 2020), (Grundmann, 2017), (Grundmann et al., 2021), (Swogger et al., 2015; Smith and Lawson, 2017; Singh et al., 2018c; Leong Bin Abdullah et al., 2021)	Some chronic, frequent kratom users report dependence/addition and/or withdrawal, but this is generally more readily self-managed compared to use disorders of other drugs of abuse	

#### 3.1.1 Summary of 2018 Findings

There were no animal intravenous drug self-administration (IV DSA), intracranial self-stimulation (ICSS) brain reward, or physical dependence/withdrawal studies of kratom’s alkaloids; however, other data suggested relatively low abuse potential as compared to opioids and other drugs of abuse (Henningfield et al., 2018a). There was evidence of morphine opioid receptor (MOR) mediated effects, and preliminary drug discrimination and conditioned place preference (CPP) studies with rats suggested abuse related effects at high intolerable human dose equivalents.

Survey data from the US and field studies in Southeast Asia (SEA) showed most kratom use was for health-related benefits, and to facilitate occupational performance. Data indicated that problem abuse and addiction were not common and was generally more tolerable and readily self-manageable as compared to opioids. A frequent reason for use was as an opioid substitute for pain and self-management of opioid, alcohol, and other drug dependence.

#### 3.1.2 Factor 1 Science Updates

##### 3.1.2.1 Intravenous Drug Self-Administration Trials

Rates of MG self-administration were similar to those of saline, and MG pretreatment produced dose-related reductions in morphine self-administration rates (Hemby et al., 2019). The authors concluded “The present findings indicate that MG does not have abuse potential and reduces morphine intake, desired characteristics of candidate pharmacotherapies for opiate addiction and withdrawal … ”. 7-OH-MG was self-administered at high doses and pretreatment increased morphine self-administration.

MG self-administration rates in rats did not exceed those obtained with saline and MG pretreatment decreased heroin self-administration, with little effect on methamphetamine self-administration (Yue et al., 2018). The authors noted “These results suggest limited abuse liability of mitragynine and the potential for mitragynine treatment to specifically reduce opioid abuse. With the current prevalence of opioid abuse and misuse, it appears currently that mitragynine is deserving of more extensive exploration for its development or that of an analog as a medical treatment for opioid abuse.” These results are consistent with human reports that kratom is useful in the management of opioid craving and withdrawal and to support opioid abstinence (Grundmann et al., 2018; Coe et al., 2019; Prozialeck et al., 2019; Garcia-Romeu et al., 2020).

##### Intracranial Self-Stimulation

In the ICSS model, rats equipped with brain electrodes self-deliver rewarding electrical brain stimulation. Opioids, amphetamine-like stimulants, cocaine, and other classic drugs of abuse reduce the stimulation threshold and increase the strength of the rewarding effects of drugs on ICSS (Negus and Miller, 2014). Neither MG nor 7-OH-MG showed evidence of brain rewarding effects, whereas morphine robustly and dose-dependently decreased the stimulation threshold (Behnood-Rod et al., 2020). Thus, the ICSS results suggest lower brain rewarding effects of MG as compared to morphine.

##### Drug Discrimination Studies

The discriminative stimulus effects of MG were evaluated in studies designed to assess generalization to morphine as well as the delta-opioid receptor agonist SNC80 and kappa-opioid receptor agonist U69593, alpha adrenergic agonists lofexidine, clonidine and phenylephrine, alpha adrenergic antagonists yohimbine and atipamezole, and the cannabinoid agonist ∆-9- tetrahydrocannabinol (Reeve et al., 2020). The strongest generalization was to lofexidine and phenylephrine, both unscheduled drugs: phenylephrine is in some over-the-counter cold medicines; lofexidine is approved for several indications including the first nonopioid for alleviating opioid withdrawal.

In a comparison of MG and 7-OH-MG across studies that included *in vitro* receptor binding and an antinociception test, MG partially generalized to morphine, whereas 7-OH-MG fully generalized to morphine in rats (Obeng et al., 2021). Similarly, Hiranita et al. (2020) found only partial generalization of oral MG to i.p. morphine in rats (Hiranita et al., 2020).

##### 3.1.2.4 Conditioned Place Preference

Various MG preparations produced mixed CPP effects with some suggesting abuse potential at high doses. A low priming injection of MG or morphine reinstated CPP after establishment with either drug, suggesting rewarding effects for both (Japarin et al., 2021). Baclofen pretreatment prevented the acquisition and expression of MG-induced CPP (Yusoff et al., 2018). CPP was achieved in mice with a high dose methanolic extract of kratom leaves (Vijeepallam et al., 2019). In a fourth study (see also Factor 2), lyophilized (freeze-dried) kratom tea (LKT), a potential treatment for pain and opioid dependence, did not induce CPP in mice (Wilson et al., 2020).

##### 3.1.2.5 Physical Dependence and Withdrawal

Discontinuation of morphine administration produced response rate disruptions indicating significant signs of spontaneous withdrawal, whereas discontinuation of MG administration did not produce significant signs of spontaneous withdrawal. Naloxone administration did precipitate response rate disruptions indicating withdrawal in both MG and morphine treated rats, however, this withdrawal effect was weaker and shorter lived in MG treated rats as compared to morphine treated rats (Harun et al., 2020). MG treatment also reduced naloxone precipitated withdrawal in animals receiving chronic morphine, consistent with human reports. Hassan, Pike, See, Sreenlivasan et al. (2020) compared the efficacy of MG to methadone for treating morphine withdrawal in rats concluding that MG treatment attenuated withdrawal symptoms significantly, similar to methadone and buprenorphine, and potentially with less undesired effects (Hassan et al., 2020).

Although MG withdrawal signs are weak in rats compared to those from morphine withdrawal, there does appear to be evidence of physical dependence; however, MG withdrawal unlike morphine was not associated with anxiogenic-like subjective symptoms. When using a pentylenetetrazol (PTZ) discrimination trial to evaluate anxiogenic signs in rats after MG or morphine withdrawal precipitated by naloxone, MG showed no substitution to the PTZ discriminative stimulus, while morphine produced a dose-related PTZ-like stimulus, further supporting MG as a novel pharmacotherapeutic intervention for managing opioid use disorder (Johari et al., 2021).

Other studies of opioid or MG withdrawal suggested that specific brain proteins might serve as more sensitive biomarkers for physiological dependence in rats as compared to behavioral signs (Hassan et al., 2021). Clonidine treatment may attenuate MG withdrawal signs in rats (Hassan et al., 2021). Another recent study employed an open-field test and an elevated-plus maze test to evaluate naloxone-precipitated withdrawal from MG as compared to morphine, and provided additional evidence confirming that MG can induce physical dependence and measurable signs of withdrawal in rats (Harun et al., 2021a). Overall, the research is consistent with human reports that kratom withdrawal is generally more modest and more readily self-manageable than that produced by opioids (e.g., 22 and as discussed in Factor 7).

##### 3.1.2.6 Real World Evidence of Abuse and Dependence

Factors 4–6 discuss the public health aspects of kratom use; however, many of the same studies address Factor 1 concerning evidence for abuse and are mentioned here.

As reported by Henningfield, et al. (2018), although surveys and anecdotal reports in the US and SEA confirm that some kratom consumers reported “addiction” those studies also indicated that use “to get high” is relatively low as compared to opioids and other recreational drugs of abuse, and that use by smoking, injecting, and/or insufflating was rare as compared to opioids, stimulants and other recreational drugs (Henningfield et al., 2018a). Recent studies confirm that kratom intake can lead to dependence and withdrawal in some kratom users, but these are substantially less likely to interfere with family, social and occupational life and commitments as compared to opioid dependence. Moreover, kratom is widely viewed as a healthier and less life-impairing substance to replace drugs such as opioids, alcohol, and stimulants (Singh et al., 2014; Galbis-Reig, 2016; Swogger and Walsh, 2018; Prozialeck et al., 2019).

A variety of reports confirm kratom use to self-manage opioid withdrawal and that abstinence from high chronic kratom use is typically associated with milder symptomatology than abstinence from classical opioids (Grundmann et al., 2018; Smith et al., 2019; Garcia-Romeu et al., 2020). The conclusion of Prozialeck et al. (2019) and Grundmann et al. (2018) (Grundmann et al., 2018; Prozialeck et al., 2019) were further strengthened by two published US surveys which found that the overwhelming majority of kratom consumers reported that their use was for various health benefits and not for recreational purposes (Coe et al., 2019; Garcia-Romeu et al., 2020; Harun et al., 2021b).

#### 3.1.3 Factor 1 Updated Conclusion

Diverse scientific approaches were employed to profile MG’s abuse potential, finding no evidence of rewarding effects in the IV self-administration and ICSS models, and weak evidence of potential reward in the CPP procedure. MG only partially generalizes to morphine and more fully generalizes to the nonscheduled alpha-adrenergic agonists, phenylephrine and lofexidine. The new data suggest relatively low abuse potential as compared to morphine-like opioids, stimulants, and other drugs of abuse that demonstrate robust rewarding effects across all such abuse potential models. Similarly, MG’s potential to produce physical dependence and withdrawal appears relatively low, but not absent, as compared to opioids in animal models. These findings are generally consistent with human reports that MG has a relatively low abuse and withdrawal potential as compared to recreationally used opioids but can reduce opioid self-administration and withdrawal. Surveys indicate that reducing opioid self-administration and withdrawal are among the most common reasons for kratom use in the US (also discussed in Factors 4, 5, and 6). New studies discussed in Factors 2–7 contribute further to the understanding of kratom’s abuse potential, including its public health risks and benefits, that are part of the 8-factor abuse potential assessment.

### 3.2 Factor 2—Scientific Evidence of its Pharmacological Effects

#### 3.2.1 Summary of 2018 Findings

MG and 7-OH-MG have some MOR mediated effects, but 7-OH-MG occurs at low concentrations in kratom leaves and is absent in many kratom product derivatives suggesting that the effects reported by kratom consumers are due primarily to MG. Some kratom effects were shown to be naloxone reversible (e.g., “pain” tolerance); however, MG and 7-OH-MG mechanisms of action were diverse and mediated by non-opioid transmitters and pathways (Kruegel and Grundmann, 2018). Thus, characterization of MG as an opioid “analog” or “narcotic like opioid” is not consistent with the overall evidence, leading Henningfield et al. (2018) to conclude “More research is clearly needed to elucidate receptor binding profiles and the diverse and probably complex mechanisms of action of the kratom alkaloids singly, in combination, and as commonly occur in marketed products and brewed extracts” (Henningfield et al., 2018a).

#### 3.2.2 Factor 2 Science Updates

##### 3.2.2.1 Potential Therapeutic Effects

Although neither kratom nor any of its alkaloids are approved for therapeutic use for any disorder, surveys discussed in *Factors 4, 5, and 6—History and Current Patterns of Abuse; the Scope, Significance and Duration of Abuse; what, if Any, Risk is There to the Public Health* and elsewhere (Henningfield et al., 2018a; Grundmann et al., 2018; Swogger and Walsh, 2018; Coe et al., 2019; Prozialeck et al., 2019; Garcia-Romeu et al., 2020) show individuals in the US and around the world describe using kratom for its health benefits. Research characterizing kratom’s effects, mechanisms of action, and therapeutic kratom alkaloid use rapidly advanced since 2018. In a placebo-controlled cold pressor task evaluating anti-nociceptive effects, pain tolerance was significantly increased following consumption of a kratom tea-type decoction similar to Malaysian preparations (Vicknasingam et al., 2020). These data provided “the first objectively measured evidence obtained in controlled research with human subjects that are preliminarily supporting or confirming previously published reports of kratom pain relieving properties based on self-reports collected in observational studies”.

Consistent with Vicknasingam et al. (2020)’s clinical findings, oral LKT administration to mice produced dose-related antinociceptive effects at doses that did not alter locomotion or produce CPP; there were brief, non-life threatening decreases in respiration (Behnood-Rod et al., 2020). Repeated LKT administration produced no physical dependence, but significantly decreased naloxone-precipitated withdrawal in morphine dependent mice, confirming MOR agonist activity and therapeutic LKT effect for treating pain and opioid physical dependence.

After investigating *in vitro* receptor binding affinity and *in vivo* morphine discrimination, antinociception in the “heated plate” pain test, and naloxone challenge tests in rats, the authors concluded “At human m-opioid receptor (MOR) *in vitro*, mitragynine has low affinity and is an antagonist … “. Overall, 7-OH-MG had stronger MOR mediated effects including antinociception (Obeng et al., 2021). An extensive series of tests characterized several minor indole and oxindole alkaloids that the authors suggest are insufficient in abundance to account for the biological effects of kratom but may show promise for the development of potential medicines including potential new chemical entities (Chakraborty et al., 2021a).

Several of these studies showed MOR mediated antinociceptive effects with little evidence of respiratory depression suggesting the potential to contribute to new generations of nonopioid analgesics.

##### 3.2.2.2 Mechanisms of Action

Although kratom produces some effects in common with opioids, and some of its alkaloid’s actions are mediated by MOR receptors, its effects and mechanisms of action are diverse and include non-opioid mechanisms of action and non-opioid acting constituent alkaloids, as discussed in earlier reviews (Henningfield et al., 2018a; Kruegel and Grundmann, 2018; Prozialeck et al., 2019). In 2021, Leon et al. (2021) investigated several alkaloids, including mitragynine, paynantheine and speciogynine that produce serotonergic effects potentially mediated by their metabolites. As the authors discuss, such actions would be consistent with some of the mood enhancing effects attributed to kratom (Kruegel and Grundmann, 2018; Sharma and McCurdy, 2021).

Kratom contains approximately 1–2% MG by weight, as well as other alkaloids (including 7-OH-MG) that typically are present at such low levels in kratom leaf material that it is uncertain if they contribute to kratom effects (Prozialeck et al., 2019). 7-OH-MG is present in low concentrations in natural kratom products, but gradually emerges *in vivo* as a MG metabolite. Kruegel et al. (2019) studied its role as a mediator of MG effects (Kruegel et al., 2019) summarizing “7-hydroxymitragynine is formed from mitragynine in mice and … brain concentrations of this metabolite are sufficient to explain most or all of the opioid-receptor-mediated analgesic activity of mitragynine … it suggests a possible explanation for the seemingly improved safety profile of mitragynine compared to classical opioid agonists … We believe mitragynine and related compounds have great potential as future therapeutics, but metabolic processes must be carefully considered as the field continues to advance.” Hiranita, Sharma, Oyola et al. (2020) reported although “the conversion rate of 7‐hydroxymitragynine from p.o. mitragynine is low, 7‐hydroxymitragynine is a more potent and efficacious μ‐opioid receptor agonist than mitragynine, suggesting that conversion to this metabolite may contribute to the *in vivo* μ‐opioid activity of mitragynine” (Behnood-Rod et al., 2020).

Kratom is commonly consumed to enhance occupational performance and as a coffee substitute for energy at low doses. In an animal model of spatial learning and memory, high doses impaired memory (Hassan et al., 2019). Suhaimi, Hassan, Mansor and Müller (2021) reported changes in brain electroencephalogram (EEG) activity after acute and chronic MG exposure in rats, with strong effects on some measures at high doses, supporting the importance of more research on brain function and potential cognitive effects (Suhaimi et al., 2021).

Gutridge et al. (2020) pharmacologically characterized interactions between kratom extracts, kratom alkaloids, and synthetic carfentanil-amide opioids with G-proteins and beta-arrestin at mu, delta and kappa opioid receptors *in vitro,* and assessed whether they had rewarding properties and the degree to which they reduced alcohol intake (Gutridge et al., 2020). The authors concluded that “kratom alkaloids do not recruit β-arrestin 2 at the μOP, δOP, and κOP and can significantly reduce both moderate and binge alcohol intake in male and female mice. This pharmacological profile and effect on alcohol intake in rodents may explain why some find kratom useful to self-medicate for alcohol use disorder.” These findings were further supported by the findings by Todd et al. (2020) who concluded “mitragynine and 7-hydroxymitragynine demonstrate functional selectivity for G-protein signaling, with no measurable recruitment of β-arrestin. Overall, the study demonstrates the unique binding and functional profiles of the kratom alkaloids, suggesting potential utility for managing pain, but further studies are needed to follow up on these *in vitro* findings” (Todd et al., 2020).

Hiranita et al. (2019) compared the effects of MG to morphine in behavioral and antinociception assays in rat models finding “Opioid receptors do not appear to mediate the disruptive effects of mitragynine on learned behavior. Mitragynine had lesser antinociceptive effects than morphine, and these did not appear to be mediated by opioid receptors. The pharmacology of mitragynine includes a substantial non-opioid mechanism” (Hiranita et al., 2019).

##### 3.2.2.3 Studies of Kratom Minor Alkaloids and Their Metabolites, and Analogs

Advances in analytical methods are accelerating our understanding of the effects of numerous kratom alkaloids including liquid chromatography–tandem mass spectrometry assays that quantify kratom alkaloids in kratom leaf extracts and commercial products (Sharma et al., 2019).

Most of these alkaloids are present at de minimis levels with respect to human experience, effects, and safety; however, it is possible that while the majority of natural plant-based kratom preparation effects are mediated by MG, one or more minor alkaloids may also play a minor role and account for differences in kratom strains (Kruegel et al., 2019; Chear et al., 2021).

An *in vitro* pharmacological characterization of five kratom based minor alkaloids found that their low abundance made it unlikely that these alkaloids play a major mediating role in the biological actions of kratom consumed by humans, but this research may contribute to furthering the understanding of kratom mechanisms of action and opioid receptor function (Chakraborty et al., 2021a).

Kratom alkaloids are of interest as templates for novel synthesized molecules (i.e., analogs) for new medicines. One third to one half of FDA-approved medicines are based on natural plant product substances from which novel chemical entities were developed (Newman and Cragg, 2016; Domnic et al., 2021a). Such efforts are actively in progress characterizing a variety of indole and oxindole alkaloids, determining their chemical structures, and binding affinities for opioid and other receptors (Chear et al., 2021). One approach to the synthesis of novel MG analogs produced several partial MOR agonists with low G-protein activation (Chakraborty et al., 2021b). These analogs demonstrated robust analgesic effects but low respiratory depressant, locomotor, and conditioned place preference suggesting lower adverse effects including abuse potential.

Combinations of kratom alkaloids may inhibit cell proliferation and migration of nasopharyngeal carcinoma cells suggesting alkaloid or new analogs as potential cancer treatments (Domnic et al., 2021b).

##### 3.2.2.4 MG Metabolism and Metabolite Profiling

Thirteen MG metabolites were identified in human liver microsomes (HLM) and S9 fraction studies (Kamble et al., 2019), and potential MG and other kratom alkaloids drug interactions were investigated including with pharmaceutic products (Kamble et al., 2020a).

7-OH-MG can be converted to pseudoindoxyl-MG in human plasma to a greater extent than is produced in mice, rats, dogs and cynomolgus monkeys, possibly explaining potential human effects and benefits that may not be predicted in animal studies alone (Kamble et al., 2020b).

#### 3.2.3 Factor 2 Updated Conclusion

Kratom’s main effects are due to the consumption of MG, but other minor alkaloids and metabolites, including 7-OH-MG, may also contribute to effects reported by consumers. Since 2018, many scientific advances improved our understanding of how these alkaloids and metabolites interact. Some alkaloids that contribute little to the effects of kratom may ultimately contribute to safer and more effective new medicines for a variety of disorders, as well as for general health and well-being. Development and approval of such products may be a decade or more in the future, but this rapidly advancing science is explaining how kratom works, and why its pain relieving, and other benefits occur with relatively low levels of abuse, dependence, and harmful decreases in respiration compared to opioids.

### 3.3 Factor 3—The State of Current Scientific Knowledge Regarding the Drug

#### 3.3.1 Summary of 2018 Findings

The 2018 8-FA highlighted kratom’s pharmacodynamic effects. Preclinical anti-nociceptive studies suggested that MG and 7-OH-MG produced such effects mediated by MOR receptors. Most information about the effects of long-term use in humans on various physiological, and cognitive parameters was based on anecdotal reports, case histories, and preliminary field studies in SEA. A two-compartment model best described human oral MG pharmacokinetics (Trakulsrichai et al., 2015).

#### 3.3.2 Factor 3 Science Updates

New kratom pharmacokinetics studies in rats, mice and dogs document plasma MG, 7-OH-MG, and other alkaloids and minor metabolites over 12 h or more, with accompanying safety assessments. Six new clinical studies following long-term kratom use provide safety data on health, and organ and brain function were also conducted.

##### 3.3.2.1 Pharmacokinetics and Pharmacodynamics Findings Related to MG and 7-OH-MG Safety

After oral administration of traditional or other natural kratom formulations to rats, greater systemic exposure was observed than that of an equivalent oral MG dose alone; no adverse events were reported (Avery et al., 2019).

Administration of 5 mg/kg oral MG (equivalent to approximately 3 mg/kg in humans) and 0.1 mg/kg IV MG to beagle dogs was well tolerated and produced no adverse events or major abnormalities in clinical parameters (Maxwell et al., 2020).

The estimated MG clearance (CL/F) was 2.21 L/h, absorption rate (Ka) 0.82/h, and volume of distribution (Vd) 30.8 L after oral 20, 40, and 80 mg/kg MG doses to rats (Jagabalan et al., 2019). Oral 55 mg/kg MG produced 85 ng/ml Cmax for 7-OH-MG, 14 times lower than the MG Cmax. Anti-nociception after IV MG and 7-OH-MG suggested that 7-OH-MG was more potent and efficacious than MG, and metabolic formation of 7-OH-MG contributes to *in vivo* MOR mediated effects of oral MG (Hiranita et al., 2020).

##### 3.3.2.2 Pharmacokinetic and Pharmacodynamic Findings With Kratom’s Minor Alkaloids

MG, 7-OH-MG, corynantheidine, speciogynine, speciociliatine, paynantheine, corynoxine, corynoxine-B, mitraphylline, ajmalicine, and isospeciofoline were analyzed in rat plasma after a variety of oral kratom products, with only MG, 7-OH-MG, speciociliatine, and corynantheidine quantifiable at 8 h (Kamble et al., 2021).

Speciociliatine pharmacokinetics were characterized following IV and oral dosing to help understand the potential contribution of this alkaloid to *in vivo* kratom administration effects (Berthold et al., 2021). Speciociliatine had higher systemic exposure and lower clearance compared to the other kratom alkaloids mitragynine and corynantheidine. Similarly, the pharmacokinetics of corynanthidine, a minor kratom alkaloid and perhaps a MOR antagonist, were determined after 2.5 mg/kg IV and 20 mg/kg oral doses to rats, yielding a 50% oral bioavailability, a 4.1 h Tmax and extensive distribution including in brain corpus callosum and hippocampus regions (King et al., 2020).

##### 3.3.2.3 Safety Assessments From Clinical Studies

Kratom’s anti-nociceptive effects in the cold pressor test are described in Factor 2 and its potential for physiological dependence and withdrawal are discussed in Factor 7 (Vicknasingam et al., 2020). This section summarizes six new clinical studies that assessed health and safety endpoints.

Leong Bin Abdullah et al. (2020) studied the lipid profiles, liver function and blood chemistries in 100 chronic kratom users and 100 healthy nonusers in Malaysia finding that the “liver parameters of the study participants were within normal range. The serum total cholesterol and LDL of kratom users were significantly lower than those of healthy subjects who do not use kratom. There were no significant differences in the serum triglyceride and HDL levels. However, higher average daily frequency of kratom use and increasing age were associated with increased serum total cholesterol among kratom users.”

Singh, Muller, Murugaiyah et al. (2018) studied various hematological and clinical-chemistry parameters of kratom users in Malaysia (Singh et al., 2018a). They interviewed and collected blood samples from 58 “regular kratom users” and 19 “healthy controls.” Findings showed there were no significant differences in the hematological and clinical-chemistry parameters of traditional kratom users and healthy controls, except for HDL and LDL cholesterol values; these were found to be above the normal reference range for the users. Similarly, long-term kratom consumption (>5 years), and quantity of daily kratom use (≥3 ½ glasses; mitragynine content 76.3–114.8 mg) did not appear to alter the hematological and biochemical parameters of kratom users. These data suggest that even long-term and heavy kratom consumption did not significantly alter the hematological and clinical-chemistry parameters of kratom users in a traditional setting.

Singh, Narayanan, Grundmann et al. (2020), studied the long-term effects of kratom use in thirteen people in Malaysia who used kratom longer than 20 years in a cross-sectional pilot study (Singh et al., 2020a). They summarized their results as follows: “Respondents were required to undergo a blood-test and laboratory analysis was conducted to determine the mitragynine content in an acquired street sample of kratom. The regular, long-term consumption of brewed kratom decoction did not cause any significant alterations in haematological, kidney, liver, thyroid, inflammatory and gastrointestinal analytes in a cohort of kratom users who had no history of substance misuse. However, those who had a higher intake (>3 glasses per day) of kratom exhibited higher lipid values (except for HDL-cholesterol), and a moderate elevation of homocysteine level. Long-term (>20 years with a daily intake of ≥87.54 mg mitragynine) kratom consumption was not associated with altered biochemical levels, although prolonged and chronic, frequent use (>3 glasses daily) may result in cardiovascular risks.” Note that this study was not designed to determine if kratom or other factors contributed to higher lipid values.

Singh, Chye, Suo et al. (2018) conducted a preliminary study of the impact of kratom use on brain function, as assessed by brain magnetic resonance imaging, among chronic kratom users in Malaysia. They reported “There were no significant differences (*p* > 0.05) in the intracranial volume (ICV), cortical volumes (frontal, parietal, temporal, occipital, or cingulate lobe), or subcortical volumes (striatum, hippocampus, or amygdala), as well as in the diffusion tensor imaging (DTI) metrics, fractional anisotropy (FA) and mean diffusivity (MD) between kratom users and the controls. This preliminary study showed long-term consumption of kratom decoction is not significantly associated with altered brain structures in regular kratom users in traditional settings” (Singh et al., 2018b).

Singh, Narayanan, Muller et al. (2019) studied potential long-term cognitive effects associated with kratom use in kratom uses in Malaysia with assessments performed using the Cambridge Neuropsychological Test Automated Battery (Singh et al., 2019a). Relative to control participants, higher consumption (>3 glasses daily or mitragynine doses between 72.5 and 74.9 mg) of kratom tea was selectively associated with impaired performance on the Paired Associates Learning task of the Cambridge Neuropsychological Test Automated Battery, reflecting deficits in visual episodic memory and new learning.

Leong Bin Abdullah, Tan, et al., evaluated the prevalence of ECG abnormalities and QTc intervals in kratom users without histories of illicit drug use. Sinus tachycardia was higher in kratom users. Daily kratom consumption was associated with borderline QTc intervals (Leong Abdullah et al., 2021). Another study by Leong Bin Abdullah and Singh found that people who consumed four or more glasses of kratom tea daily had higher MG concentrations than lower intake consumers and this higher intake was associated with prolonged QTc intervals (Leong Bin Abdullah and Singh, 2021a). The same authors published a comprehensive review of the cardiovascular and cardiotoxic effects of kratom and came to the conclusion that limitations in studies to date do not permit definitive conclusions about the cardiovascular risks (Leong Bin Abdullah and Singh, 2021b).

#### 3.3.3 Factor 3 Updated Conclusion

Pharmacokinetics and safety data from multiple species, kratom preparations, alkaloids, and metabolites; advances in bioanalytical assays providing more accurate and reliable findings; and data from multiple studies with MG doses many times higher than those human kratom users take are now available. These studies add to those described in Factors 1 and 2 confirming little evidence of serious adverse or life-threatening effects over a broad range of doses, dosage forms, and in four species (mouse, rat, dog, and monkey).

Other major advances in kratom science come from six clinical studies of long term kratom use effects and safety, as well as the study of anti-nociceptive effects of kratom and physiological dependence described in Factors 2 and 7. These important advances in kratom science evaluated the effects of long-term kratom use on a variety of physiological parameters including kidney and liver function, hematological parameters, cognition, and on brain function by brain magnetic resonance imaging. Although relatively small studies, none suggest serious adverse consequences of use. It is important to note that these are not definitive safety studies and cannot be used to claim that kratom has no adverse effects on any of the studied physiological domains and limitations of each study were noted in the publications. Nonetheless, the findings are encouraging and should facilitate the conduct of more comprehensive follow-up studies.

### 3.4 Factors 4, 5, and 6—History and Current Patterns of Abuse; the Scope, Significance and Duration of Abuse; what, if Any, Risk is There to the Public Health

#### 3.4.1 Summary of 2018 Findings

Note that for this update, Factors 4, 5, and 6 are considered together because they all contribute to understanding nonmedical use, recreational use and abuse, and public health impact, relying on some of the same surveys across factors. The Henningfield et al., 2018 8-FA considered all major relevant federal surveys, as well as data from internet monitoring, and more than 20,200 comments to the DEA, and concluded that there was no evidence of an imminent public health threat associated with kratom (Henningfield et al., 2018a). To the contrary, the review concluded that there were foreseeable health risks including opioid overdose and deaths if lawful kratom was banned and possession criminalized. Moreover, although kratom is not approved as safe and effective for therapeutic use, it was evident that most kratom use in the US was for health and well-being by people who personally found kratom to be more effective, tolerable, accessible and/or preferred a natural product as compared to FDA approved medicines.

#### 3.4.2 Factors 4, 5, and 6 Science Updates

##### 3.4.2.1 U.S. National and Federal Survey Data

Table 2 summarizes the main findings from the major national and federal surveys and other data sources. Overall, there are more similarities than differences with respect to demographics reported in this table as well as in other demographics reported in the published survey results. Prevalence appears to be substantially underestimated by the NSDUH and RADARS surveys (U.S. Department of health and Human Services, 2020; Schimmel et al., 2021).

**TABLE 2:** Summary of data sources.

Survey/Data source	Main results and comment	Other comments
Drug Abuse Warning Network (Drug Abuse Warning Networ, 2020)	No reports in DAWN from 1970 to 2011	
	“New DAWN” began in 2019 and has not listed kratom	
Monitoring the Future Study (Miech et al., 2021)	Kratom use is not assessed	Note that 9% of NSDUH Reports were from age 12–17 year olds
National Forensic Laboratory Information Service (Drug Enforcement Adm, 2020a)	Since 2016 NFLIS did not include MG/kratom reports because the rates are below the threshold for inclusion	
National Survey on Drug Use and Health (U.S. Department of health and Human Services, 2020)	Paid responders on national panel (*n* = 67,625).^6^	See Grundmann et al., 2021 and Henningfield et al., 2021 comment on apparent underestimation of kratom use prevalence (Grundmann et al., 2021; Henningfield et al., 2021)
	2019 Prevalence Lifetime Use: 1.4%; Past Year Use: 0.7%	
Treatment Episodes Data Set (Substance Abuse and Menta, 2020)	No reports. This does not mean there were no reports but suggests subthreshold signal	Internet chatrooms and SUD treatment clinic advertising suggests some kratom users are seeking cessation assistance
Coe et al. (2019) (Coe et al., 2019)	Internet Survey of self-identified kratom users age ≥18 (*n* = 2,867)	
	48% use for self-management of pain	
	10% for self-management of opioid UD or withdrawal	
	22% use for mood management	
	2.4% use to get high	
Garcia-Romeu et al. (2020) (Garcia-Romeu et al., 2020)	Internet Survey of self-identified kratom users, age ≥18 (*n* = 2,798)	2% met DSM-5 criteria for past-year moderate or severe kratom-related SUD, but it was rated very low on scale of concern and adverse impact
	91% use for self-management of pain	
	41% for self-management of opioid UD or withdrawal	
	67% for management of anxiety	
	65% for depression	
	<3% report kratom dependence	
Covvey et al. (2020) (Covvey et al., 2020)	Nationally representative Internet survey of persons aged 18–59 (*n* = 1842)	Similar demographics as Grundmann 2017, Coe et al., 2019 and Garcia-Romeu et al., 2020 but may have underestimated % over 50 due to 59 year old upper age limit of survey. ((Coe et al., 2019), (Garcia-Romeu et al., 2020), (Grundmann, 2017)) Reasons for use were not asked, e.g., to self-manage pain, addiction, mood
	112 (6%) reported lifetime kratom use	
	72% were 25–44 years old, male, employed, and at higher educational levels	
	24–47% of respondents indicated self-reported diagnoses for any addiction, and 43% reported previously received treatment for addiction	
Schimmel et al. (2021) (Schimmel et al., 2021)	RADARS^®^ survey of paid survey responder on national panel age >18 (*n* = 59,714)	Reasons for use were not asked, e.g., pain, addiction, mood. See Grundmann et al., 2021 and Henningfield et al., 2021 comment on apparent under estimation of kratom use prevalence (Grundmann et al., 2021; Henningfield et al., 2021)
	0.8% lifetime use	
	44% age >35	
	61% male	
	59% past year opioid use	

NSDUH, RADARS, and Covvey et al. did not report reasons for use; however, many kratom users reported past or present use of opioids and/or drug addiction treatment consistent with past findings that self-management of addiction and withdrawal is a common reason for kratom use (National Institute on Drug Abuse, 2019; Coe et al., 2019; Garcia-Romeu et al., 2020; U.S. Department of health and Human Services, 2020; Schimmel et al., 2021; Covvey et al., 2020; Grundmann, 2017). Survey data incidence reports for DAWN, MTFS, NFLIS, and TEDS are apparently below the threshold for reporting as confirmed in an inquiry to NFLIS (Drug Enforcement Administration, 2020a; Drug Abuse Warning Network, 2020; Substance Abuse and Mental, 2020).

These findings do not support the conclusion that kratom use represents an imminent health threat and in fact kratom is not listed in the most recent DEA National Drug Threat Assessment (Drug Enforcement Administration, 2020b). There is no evidence that kratom is “fueling” or otherwise contributing to the opioid epidemic, though the survey data suggest that it is an informal self-management approach supporting the efforts of many opioid users to reduce and discontinue opioid use (Grundmann, 2017; Coe et al., 2019; Garcia-Romeu et al., 2020; Grundmann et al., 2021).

##### 3.4.2.2 Kratom Use Prevalence

As mentioned in Table 2, the NSDUH and RADARS surveys may greatly underestimate the US prevalence and incidence of kratom use, with estimates of past year kratom use of 1,790,00–2,040,000.^3^ (U.S. Department of health and Human Services, 2020; Schimmel et al., 2021). In contrast, a credible estimate based on market data suggested prevalence of 3–5 million in 2014–2015 (Botanical Education Allia, 2016).

Experts and marketers agree that the kratom market substantially expanded since that time, with kratom export data from Indonesia to the US and major marketer consensus finding that the US consumer base was likely 10–16 million. This is consistent with a nationally projectable survey estimate from 2020 concluding past year kratom use as 4.1% or 10,500,000 kratom users (Covvey et al., 2020).

##### 3.4.2.3 Kratom Use Associated Mortality

The two most widely cited estimates of kratom associated mortality are based on world-wide reports over nearly 10 years (Food and Drug Administration, 2018; Olsen et al., 2019). FDA’s statement noted that all but one involved other substances, and that case was under further investigation.^4^ Medical examiners or coroners reported kratom as the cause of death in 91 (59.9%) of 152 kratom positive decedents (out of 27,338 overdose deaths in 27 states), including seven for whom kratom was the only substance positive on postmortem toxicology, although other substances could not be ruled out (Olsen et al., 2019). In approximately 80% of kratom positive or “involved” deaths, decedents had a history of “substance misuse”, with 65% of cases listing fentanyl as the cause of death, 32.9% heroin, followed by benzodiazepines, prescription opioids, and cocaine. An earlier study (Gershman et al., 2019) cautioned that comprehensive toxicology might identify other substances contributing or causing death. We are not aware that any of the 93,000 drug overdoses estimated for 2020 included deaths due to kratom. That does not mean that there were no deaths in which kratom was the primary cause or a contributing factor; however, the signal is clearly low.

An assessment of various survey data concluded that the risk of kratom associated death was at least a thousand times lower than for morphine-like opioids (Henningfield et al., 2019). This is consistent with NIDA’s position (National Institute on Drug Abuse, 2019) and with the 2018 DHHS kratom scheduling rescission letter and the conclusions drawn by Assistant Secretary of Health Brett P. Giroir, MD, ADM who stated: “There is still debate among reputable scientists over whether kratom by itself is associated with fatal overdoses” (Giroir, 2018).

##### 3.4.2.4 Mortality Risks Projected as a Result of Banning Licit Kratom

Surveys and more than 20,000 comments to the DEA suggest that many kratom users fear resumption of opioid use and the need to resort to illicit kratom markets (Drug Enforcement Adm, 2016; Grundmann, 2017; Coe et al., 2019; Garcia-Romeu et al., 2020). It is not possible to project how many people would relapse to opioids and potentially overdose (Henningfield et al., 2018a; Henningfield et al., 2018b; Henningfield et al., 2018c; Henningfield et al., 2018d; Grundmann et al., 2018; Prozialeck et al., 2020). This was a concern of the DEA in withdrawing its 2016 kratom scheduling proposal (Ingraham, 2016b) and in the US DHHS kratom scheduling recission letter (Giroir, 2018).

##### 3.4.2.5 Public Health and Individual Benefits of Kratom

In 2018, a systematic review of kratom use and mental health by Swogger and Walsh concluded “…kratom use appears to have several important mental health benefits that warrant further study. Kratom dependence is a risk for some people, though the dependence syndrome appears to be mild in its psychosocial and physiological effects relative to that of opioids. More and better research, including well-controlled, prospective studies, is necessary to further elucidate kratom’s potential for good and harm and the moderators of its effects” (Swogger and Walsh, 2018). The therapeutic potential of kratom based on surveys, anecdotal reports, and nonclinical research supports the plausibility of such benefits as discussed by other reviewers (Prozialeck et al., 2019; Hemby et al., 2019; Yue et al., 2018; Grundmann et al., 2018; Kruegel and Grundmann, 2018; Sharma and McCurdy, 2021; Swogger et al., 2018; Prozialeck et al., 2021).

The most important public health benefits with respect to mortality are widely agreed upon by kratom experts and surveys, and that is its use to self-manage opioid and other drug addiction and withdrawal symptoms, and thereby reduce use and overdose from far deadlier substances. This type of use is not unique in the US but was long reported in SEA (Raffa, 2014; Henningfield et al., 2018a). This was also reported in the first major US Internet survey of kratom use (Grundmann, 2017), as well as in subsequent surveys (Coe et al., 2019; Garcia-Romeu et al., 2020; Pain News Network (2018)). This was also a conclusion of a systematic review of 13 studies addressing kratom use and mental health in the US, SEA, and other countries and regions of the world, and a review by an international consortium of kratom researchers (Swogger and Walsh, 2018; Prozialeck et al., 2019).

While the opioid epidemic represents a highly visible and deadly epidemic in its own right, it is important to recognize that many millions use kratom as their preferred approach to managing other life-threatening disorders including pain, depression, anxiety, post-traumatic stress, fibromyalgia and more (Drug Enforcement Adm, 2016; Grundmann, 2017; Coe et al., 2019; Garcia-Romeu et al., 2020).

##### 3.4.2.6 Kratom Use for Managing Opioid Use/Withdrawal and Other Health Reasons

In the first half-year of the COVID-19 pandemic, there was uncertainty about kratom supply by vendors and consumers, however, overall US supply was not affected. The main reasons for kratom use are pain relief (48%), anxiety, “PTSD” or depression (22%), increase energy or focus (10%), and “help cut down on opioid use and/or relieve withdrawal” (10%) (Coe et al., 2019). Side effects were generally minor, e.g., stomach upset, rarely required medical attention and rates and severity of “bad reactions” were generally similar to those reported by Grundmann (Grundmann, 2017).

Field studies with face-to-face interviews in Malaysia provide complementary evidence to US Internet surveys regarding reasons for use and potential benefits (Singh et al., 2019b). Motives related to mood and other factors in 116 regular kratom users employed the Drinking Motives Questionnaire (DMQ) to measure motives for kratom use, reported “heavy” kratom use as drinking more than three glasses daily (estimating that 1 glass contains 48.24–50.4 mg of mitragynine), with use associated with significantly higher means scores on the Coping and Enhancement scales. A field face-to-face survey of 92 long-term male kratom users found that 72 participants (78%) reported using kratom to “enhance sexual performance” and all but one found did their sexual performance did improve. Interestingly, among participants who described kratom intake for other reasons, 35% reported enhanced sexual performance (Singh et al., 2020b).

Patterns and reasons for use and demographics were investigated in 142 current and 62 former opioid polydrug users in Malaysia (Singh et al., 2020c). The alkaloid content of a kratom street sample was primarily MG, followed by paynantheine, speciociliatine, speciogynine, and “low levels” of 7-OH-MG. There were no significant differences in demographic characteristics between current and former opioid polydrug users except with respect to marital status, with current kratom users having a higher odds ratio of being single. While both current and former opioid users reported using kratom to ameliorate opioid withdrawal, current users had significantly higher likelihood of using kratom for that purpose; however, former opioid users were more likely to use kratom for mood elevating effects.

##### 3.4.2.7 Comment on Therapeutic Use in Context of FDA Standards

It is important to note that the benefits documented in published surveys do not constitute the basis for therapeutic claims and no kratom product or kratom alkaloid is approved for therapeutic use in the US. The FDA and other federal agencies state that there is no proven therapeutic use for kratom despite evidence that millions of people in the US and many more in SEA use kratom primarily for therapeutic, beneficial use. That evidence includes peer reviewed surveys and field studies in the US and SEA, clinical and preclinical studies showing that MG’s mechanisms of action are consistent with such effects. Moreover, several animal models used to predict efficacy for treating opioid use disorder, opioid withdrawal and pain demonstrated efficacy.

None of this research meets FDA’s standard for therapeutic efficacy that is determined by evaluation of a New Drug Application (NDA). The NDA must be supported by “substantial evidence of effectiveness,” and is defined as “evidence consisting of adequate and well-controlled investigations” (Katz, 2004; Dabrowska and Thaul, 2018). The time and cost to develop and achieve FDA approval of a product as therapeutically effective and acceptably safe varies widely but is often approximated at 10 years and 1 billion dollars (DiMasi et al., 2016; Wouters et al., 2020). Only two botanical substances, Veregen^®^ (sinecatechins) and Mytesi™ (crofelemer), were developed as drug products consistent with FDA’s Botanical Drug Guidance and both are available only as prescription drugs that is typical of new drug approvals (Food and Drug Admini, 2016).

#### 3.4.3 Factor 4, 5, and 6 Updated Conclusions

The most important finding from new US survey evidence is that the conclusion that kratom products and kratom’s primary active alkaloid, MG, pose a “serious imminent threat to public health” is not supported. This extensive survey update agrees with the Henningfield et al. (2018) conclusion: “There has been no documented threat to public health that would appear to warrant emergency scheduling of the products and placement in Schedule I of the CSA carries risks of creating serious public health problems…. Although kratom appears to have pharmacological properties that support some level of scheduling, if it was an approved drug, placing it into Schedule I, thus banning it, risks creating public health problems that do not presently exist” (Henningfield et al., 2018a).

The evidence shows that millions of people in the US purchase and use kratom products for improving health and are preferred to FDA approved medicines because for them, kratom products are more effective, accessible, and tolerable. Furthermore, many prefer managing health problems with natural products.

For those using kratom products in place of opioids, which appears to be approximately 1/3 of all kratom users, it is foreseeable that removing kratom from the legal marketplace would put many at risk of returning to opioid use and risking opioid overdose death. This was clearly stated in comments to the DEA and public hearings as reported in the 2018 8-FA, and in surveys. Assistant Secretary Dr. Giroir noted “… there is a significant risk of immediate adverse public health consequences for potentially millions of users if kratom or its components are included in Schedule I, such as: … Kratom users switching to highly lethal opioids, including potent and deadly prescription opioids, heroin, and/or fentanyl, risking thousands of deaths from overdoses and infectious diseases associated with IV drug use … ” (Giroir, 2018).

### 3.5 Factor 7—The Psychic or Physiological Dependence Liability

#### 3.5.1 Summary of 2018 Findings

Recently, psychic dependence is referred to simply as “dependence” or “substance use disorder” and more commonly as “addiction” though definitions of addiction vary widely (American Psychiatric Asso, 1994; World Health Organization, 1994). Physiological dependence is often used interchangeably with the most common measure of physiological dependence, namely “withdrawal” which is also considered a clinical disorder (American Psychiatric Asso, 2013).

In the 2018 8-FA, Henningfield, Fant and Wang (2018) concluded “There have not been laboratory studies of physical or psychological dependence or abuse potential in humans caused by kratom.” Nor had classic animal studies employing the drug self-administration and physical dependence/withdrawal model been conducted (see Factor 2 in this report)”.

Nonetheless, the real-world evidence in the published literature supported the following conclusions: “…abrupt discontinuation [of kratom use] may be accompanied by withdrawal symptoms that are qualitatively similar but generally weaker than those observed following discontinuation of opioids. However, such reports make it difficult to disentangle the emergence of preexisting symptoms that had been mitigated by kratom use from those that occur as a physiological rebound accompanying the abrupt discontinuation of kratom use in kratom-dependent people. More studies of kratom’s potential to produce physical dependence, tolerance, and withdrawal are needed to characterize the nature and severity, and determinants of abstinence-associated symptoms.”

#### 3.5.2 Factor 7 Science Updates

In addition to the animal laboratory studies predictive of abuse potential, dependence, and withdrawal summarized in Factor 1, there are several new studies, surveys, and expert reviews addressing the risk and factors associated with dependence and withdrawal. A major category of kratom use is related to the typically mild and tolerable dependence and withdrawal that occurs in some frequent kratom users and the resulting use of kratom as an approach to self-management. In this context, kratom provides a harm reduction alternative to opioids in particular, but also potentially for alcohol, methamphetamine, and other drugs.

Dependence and withdrawal were addressed in a systematic review of kratom use for mental health reasons that concluded “Kratom dependence is a risk for some people, though the dependence syndrome appears to be mild in its psychosocial and physiological effects relative to that of opioids … kratom use does not appear to result in significant respiratory depression” (Swogger and Walsh, 2018). A major category of kratom use globally was to self-manage substance use disorders, consistent with the findings discussed in Factor 1 that demonstrated low abuse and physical dependence potential, and that MG administration reduced morphine and heroin self-administration, and withdrawal signs (Hemby et al., 2019; Harun et al., 2021b).

The Vicknasingam et al. (2021) study included in Factor 2 also assessed potential withdrawal signs using the Clinical Opiate Withdrawal Scale (COWS), comparing scores when participants were administered placebo or a kratom concoction (Vicknasingam et al., 2020). Although this study was not designed to be a definitive withdrawal assessment study, and did not include an opioid comparator, it was likely that people using kratom multiple times per day for many years would have experienced pronounced withdrawal symptoms. The authors concluded “None of the participants reported withdrawal symptoms either using spontaneous self-report or had significant withdrawal symptoms based on the COWS scores... All participants reported long histories of daily kratom consumption, with high frequency of daily consumption and substantial amounts consumed. It is not possible to quantify these reports into markers that could be used to approximate amounts of plant material or active ingredients consumed. However, despite the reported long duration and high levels of daily kratom consumption, during documented kratom discontinuation lasting from 10 to 20 h, no participant reported or displayed discomfort, symptoms, or signs of potential withdrawal symptoms.”

100 long term kratom users and 100 non-users in Malaysia were interviewed to assess potential symptoms related to kratom dependence and withdrawal (Leong Bin Abdullah et al., 2021). Kratom use longer than 6 years and 3 or more times per day were more likely to be associated with dependence, reduced quality of life and/or withdrawal symptoms when kratom use is discontinued. However, the authors noted that the study did not allow causative conclusions as to whether quality of life reductions are a result of increased kratom use or if such quality of life and other demographic factors contribute to more frequent kratom use.

An internet survey assessing reasons for use and effects of use in 2,798 present and past kratom users included questions about kratom dependence, withdrawal symptoms associated with discontinuation, and use to self-manage opioid dependence (Garcia-Romeu et al., 2020). Kratom-related withdrawal symptoms were reported by 9.5% of respondents with another 17.5% reporting possible kratom-related withdrawal. This supports results of previous studies (Swogger et al., 2015; Grundmann, 2017; Smith and Lawson, 2017; Coe et al., 2019) by suggesting that kratom has a relatively benign risk profile compared to typical opioids, with only a minority of respondents endorsing kratom-related adverse effects, withdrawal symptoms, or problematic use.

Coe et al. (2019) conducted an internet survey (2,867 current, 157 former kratom users) that included similar questions as Garcia-Romeu et al. and Grundmann (2017) (Grundmann, 2017; Garcia-Romeu et al., 2020), related to opioid use and effects. Kratom use was less likely to interfere with social, family, and occupational functioning compared to conventional opioids. Kratom was used by many to reduce or completely replace prescription and nonprescription opioid withdrawal and was generally considered “very effective” for managing opioid withdrawal. Relief of anxiety (including associated with post-traumatic stress disorder), depression, as well as to increase focus or energy were other major reasons for use. The foregoing conclusions are also consistent with those of Grundmann, Babin, Henningfield et al. (2021) who stated: “Some user reports suggest that regular kratom consumption carries risks of dependency and addiction, though with generally self-manageable withdrawal” (Grundmann et al., 2021).

Singh, Narayanan, Muller et al. (2018) employed widely used psychiatric instruments (Beck Depression Inventory and Beck Anxiety Inventory) to assess potential symptoms of anxiety and depression that may accompany abrupt discontinuation of kratom use in apparently frequent chronic kratom consumers in Malaysia (Singh et al., 2018c). Most respondents (70%) experienced symptoms of mild anxiety, while 81% reported symptoms of mild depression during kratom cessation. Those who consumed higher quantities of kratom tea daily (≥4 glasses) had “higher odds of reporting longer duration of kratom use history … , higher frequency of daily kratom use (≥4 times), … and were more likely to experience moderate symptoms of depression during kratom cessation” than those who consumed less. Cessation from regular and long-term kratom tea consumption was not associated with symptoms of high anxiety or depression.

#### 3.5.3 Factor 7 Updated Conclusion

Kratom’s potential to produce psychic dependence (aka “dependence” or “use disorder”) and physiological dependence (aka, “withdrawal”) advanced considerably due to surveys, and preclinical and clinical studies. Several surveys in the US, field studies in Malaysia, and a clinical trial of pain relief efficacy that included assessment of withdrawal support the conclusions of the 2018 8-FA (Henningfield et al., 2018a). Some kratom users report dependence/addiction and/or withdrawal with a greater likelihood with higher levels of chronic daily consumption. In general, it is more readily self-managed and less likely to interfere with occupational, social and family activities and responsibilities compared to dependencies to opioids, alcohol, stimulants and other drugs of abuse.

It is also important to note that there is wide individual variability, and some people do experience what they consider to be strong addiction and withdrawal to kratom. At present, it appears likely that many if not most individuals had prior histories of dependence to opioids and/or other drugs. Their conditions remain of concern nonetheless and is another area warranting further study. People for whom kratom use is considered a serious problem should have the same access to treatment as anyone with a substance use disorder. Many addiction treatment providers already advertise and offer kratom use disorder treatment assistance.

Use of opioids such as methadone and buprenorphine should be judicious in people seeking help to manage their kratom use disorder and/or withdrawal. If they formerly or are perhaps still using opioids, then the possibility of treatment with buprenorphine or methadone may be more helpful and appropriate if kratom is not satisfactory. However, for people without prior histories of recreational opioid use and dependence, treating with buprenorphine or methadone may introduce individuals to opioids and may not be the best option. This could be like treating unwanted caffeine dependence with amphetamine to replace the caffeine.

## 4 Discussion and Conclusion

In 2018, there was sufficient evidence to conclude that there was no imminent public health threat nor high degree of pharmacological abuse potential that would justify scheduling, taking into consideration the serious foreseeable adverse public health consequences of thousands of former opioid users returning to opioids and risking overdose, as well as the *de facto* criminalization of millions of US citizens. Approximately 8 months after the Henningfield et al. 8-FA was published, the US DHHS came to the same conclusion and rescinded the 2017 recommendation to place MG and 7-OH-MG in Schedule I of the CSA (Giroir, 2018). Since January 2018, there was remarkable research relevant to the abuse potential and safety of kratom from the perspective of the CSA eight factors.

Two intravenous drug self-administration studies showed that MG did not substitute for morphine (Hemby et al., 2019) or heroin (Yue et al., 2018), and that MG pretreatment reduced morphine and heroin self-administration. An intracranial brain self-stimulation (ICSS) study showed that whereas morphine produced robust decreases in the brain stimulation threshold, MG and 7-OH-MG did not (Behnood-Rod et al., 2020).

In the evaluation of physical dependence and withdrawal potential, four studies showed MG did not carry morphine-like physical dependence or withdrawal potential (Harun et al., 2020; Hassan et al., 2020; Wilson et al., 2020; Johari et al., 2021). Moreover, MG pretreatment of animals reduced spontaneous morphine withdrawal (Hassan et al., 2020). In MG physically dependent animals, withdrawal signs were qualitatively different and much weaker than morphine, consistent with its mixed mechanisms of action (Johari et al., 2021). In a mouse physical dependence model (Wilson et al., 2020), naloxone precipitated withdrawal in morphine- but not MG LKT-maintained animals, while LKT pretreatment significantly reduced withdrawal in the morphine-maintained mice.

These findings are consistent with new US survey data showing relatively low self-reported kratom addiction rates, with most people describing MG use to manage pain, depression, anxiety, opioid and other drug use disorders and withdrawal, and to increase alertness, focus and work performance. In addition, kratom dependence and withdrawal are generally weaker and more readily self-managed relative to opioids.

Extensive *in vitro* and *in vivo* animal neuropharmacology studies of the mechanisms of action of MG, 7-OH-MG and other alkaloids illustrate that they are not appropriately designated as opioids, opioid analogs, or “atypical opioids,” though some are partial agonists with low potential to recruit beta arrestin and produce respiratory depression. 7-OH-MG produces stronger MOR mediated opioid effects on abuse potential related measures and antinociception, but naturally occurs at levels so low as to not contribute meaningfully to kratom effects. This supports recommendations that regulations should prohibit kratom products with 7-OH-MG concentrations greater than occur safely in nature.^5^

Safety assessments in pharmacokinetic and pharmacodynamic studies confirm that kratom based extracts and individual alkaloids at far higher doses than consumed by humans do not appear to carry substantial mortality risk, with one analysis suggesting a mortality risk at least 1000 times less than illicit opioids (Henningfield et al., 2019). Results support the US DHHS conclusion that “experts disagree on whether kratom by itself causes overdose deaths” (Giroir, 2018; National Institute on Drug Abuse, 2019). This does not imply that kratom does not carry a mortality risk—most substances do under certain conditions and exposure levels, another important area for further research.

As to the question of whether or not kratom poses an imminent public health threat, no analysis of factors 4–6 of the 8 CSA factors, including the FDA analysis (Food and Drug Administration, 2017b), revealed kratom to pose an imminent public health risk. The US has the most comprehensive survey data to address the need for temporary or “emergency” placement of substances into CSA Schedule I. Yet none of the major surveillance systems identified such a public health threat. This includes the old and new Drug Abuse Warning Network, Monitoring the Future, National Survey on Drug Use and Health, RADARS^®^, or the Treatment Evaluation Data Set. DEA’s National Forensic Laboratory Information System mentioned kratom reports from 2010–2016 but none thereafter because the signal remained low. Neither has kratom been included in any DEA Annual National Drug Threat Reports.

The primary public health consequences of kratom use are well documented by four surveys of more than 20,000 kratom consumers summarized in this review, by Henningfield et al., 2018 (Henningfield et al., 2018a), and more than 20,000 comments to DEA (Drug Enforcement Adm, 2016) suggesting that millions of US citizens use kratom for health and well-being and many to self-manage opioid and other drug withdrawal and use disorders as their preferred approach. Many kratom users believe kratom is more effective, tolerable and/or accessible than other pharmaceuticals (Grundmann et al., 2018; Swogger and Walsh, 2018; Prozialeck et al., 2019; Prozialeck et al., 2020).

There are problems with kratom product purity (e.g., Prozialeck et al., 2020) (Prozialeck et al., 2020) and adulteration (Prozialeck et al., 2019) in the consumer marketplace. A scheduling imposed kratom ban would likely worsen these problems because kratom marketing would not discontinue and consumer demand would not cease, rather marketing would switch from regulatable lawful to illicit kratom suppliers. More states and ideally the US federal government could address these issues by product performance standards and regulatory approaches guided by science and informed through a federal rule-making approach.

Remarkably, as discussed in several reports (Henningfield et al., 2019; Prozialeck et al., 2019; Henningfield et al., 2021), there has yet to emerge a generally accepted estimate of the number of current US kratom consumers, which current ranges from approximately 2 to more than 10 million (see factors 4–6 and Henningfield et al., 2021) (Henningfield et al., 2021). As noted by Henningfield et al., 2018 and bluntly stated in the US DHHS scheduling rescission letter (Giroir, 2018), surveys need to address such issues before any action to ban consumer kratom sales and possession is contemplated. As stated in the DHHS letter:

“Further analysis and public input regarding kratom and its chemical components are needed before any scheduling should be undertaken. It is important that we have additional information to justify scheduling, such as:

• A scientific assessment of how many Americans utilize *kratom,* and an understanding of the geographic and demographic distribution of these users (Factors 4, 5);
• A scientific assessment of the actual scale and degree of dependence and/or addiction of Americans utilizing *kratom* (Factors 1, 5, 7);
• A scientific determination based on data whether *kratom* actually serves as a gateway drug that promotes further use of more dangerous opioids (Factors 1, 4, 5).
• A valid prediction of how many kratom users will suffer adverse consequences if kratom is no longer available, including among people with intractable pain, psychological distress, risk for suicide; and/or people who might transition to proven deadly opioids such as prescription opioids, heroin, or fentanyl.
• A scientifically valid assessment of causality in the current few deaths in which *kratom* was co-utilized with known lethal drugs such as fentanyl (Factors 1, 2, 3, 5 and 6)” (Giroir, 2018).

By law, scheduling considers diverse evidence including chemistry and pharmacology, level of abuse potential, physiological dependence determined in animal and human studies, as well as assessment of individual and public health risks and benefits. Taking all of these factors into account, this review provides stronger evidence than was available to Henningfield et al., or the US DHHS in 2018 (Henningfield et al., 2018a; Giroir, 2018) to recommend not only that CSA scheduling is not warranted but that CSA scheduling carries a substantial foreseeable risk of thousands of opioid overdose deaths as well as depriving millions of US citizens of one of their preferred health management assets. The fact that possession of kratom by millions of US citizens would be criminalized as a heroin-like drug felony offense is not a CSA consideration but should not be ignored.

In conclusion, we do not recommend scheduling kratom or any of its alkaloids in the CSA. We do recommend accelerated research to address the many questions raised in this review, including support of the potential development of new medicines with potential better safety and/or efficacy profiles for a variety of diseases. Finally, we recommend that the US federal government and other nations consider approaches to kratom regulation as are presently being pioneered in five US states.

## Footnotes

1 The authors welcome communications from readers on abuse‐potential and safety related kratom research published since 2018 that we might have missed.

2 For formal FDA submissions Factors 4, 5, and 6 are considered separately (see Henningfield et al., 2018a and Johnson, Griffiths, Hendricks and Henningfield, 2018 as examples), however, for temporary (also known as “emergency”) scheduling, determining if a substance poses an imminent health risk is based on the analysis of all three factors combined similarly to our approach in this review.

3 Note in a summary of RADARS data presented a few months after the Schimmel et al., 2020 publication, it was reported that the national projected past year prevalence estimate was 3.35 million.

4 FDA never reported the results of that investigation, however, the US DHHS review that led to the 2018 withdrawal of the 2017 MG and 7-OH-MG CSA scheduling recommendation determined that the incident in question was an automobile crash not attributable to kratom use.

5 Five states (AZ, GA, NV, OK, and UT) have taken this approach in their kratom consumer protection regulations and law but setting actual performance standards to address the variety of kratom based products would be seem best done by FDA which has extensive experience in such matters and could take a federal rule making approach that ensures input from diverse stakeholders representing science, public health, consumers, and the industry that prepares and manufactures kratom products.

6 Prior to 2019, NSDUH did not include kratom/MG-specific items. From 2010 through 2018, there were a total of nine (International Narcotics C, 2020a) lifetime kratom mentions (unweighted—not nationally representative), although five of those were in the last 2 years (2017 and 2018). By contrast, and over the same time frame, lifetime mentions (unweighted) of oxycodone, heroin, cocaine, amphetamine, marijuana, and other prototypic substances of abuse were in the many thousands. Lifetime aspirin mentions ranged from 7 to 23 per year, while lifetime diphenhydramine mentions ranged from 11 to 46 per year.
